# Depolymerized Fractions of Sulfated Galactans Extracted from *Gracilaria fisheri* and Their Antibacterial Activity against *Vibrio parahaemolyticus* and *Vibrio harveyi*

**DOI:** 10.3390/md20080469

**Published:** 2022-07-23

**Authors:** Manoj Tukaram Kamble, Tawut Rudtanatip, Chumporn Soowannayan, Boottoh Nambunruang, Seema Vijay Medhe, Kanokpan Wongprasert

**Affiliations:** 1 Department of Anatomy, Faculty of Science, Mahidol University, Bangkok 10400, Thailand; maav.manya@gmail.com (M.T.K.); nboottoh@gmail.com (B.N.); 2 Department of Anatomy, Faculty of Medicine, Khon Kaen University, Khon Kaen 40002, Thailand; tawut@kku.ac.th; 3 National Center for Genetic Engineering and Biotechnology, and Centex Shrimp Chalermprakiat Building, Faculty of Science, Mahidol University, Bangkok 10400, Thailand; chumporn@biotec.or.th; 4 Department of Food Chemistry, Institute of Nutrition, Mahidol University, Salaya, Phutthamonthon, Nakhon Pathom 73170, Thailand; seemamedhe@gmail.com

**Keywords:** low-molecular-weight sulfated galactans, *Gracilaria fisheri*, hydrogen peroxide, antibacterial activity, *V. parahaemolyticus*, *V. harveyi*

## Abstract

Various seaweed sulfated polysaccharides have been explored for antimicrobial application. This study aimed to evaluate the antibacterial activity of the native *Gracilaria fisheri* sulfated galactans (NSG) and depolymerized fractions against the marine pathogenic bacteria *Vibrio parahaemolyticus* and *Vibrio harveyi*. NSG was hydrolyzed in different concentrations of H_2_O_2_ to generate sulfated galactans degraded fractions (SGF). The molecular weight, structural characteristics, and physicochemical parameters of both NSG and SGF were determined. The results revealed that the high molecular weight NSG (228.33 kDa) was significantly degraded to SGFs of 115.76, 3.79, and 3.19 kDa by hydrolysis with 0.4, 2, and 10% H_2_O_2_, respectively. The Fourier transformed spectroscopy (FTIR) and ^1^H− and ^13^C−Nuclear magnetic resonance (NMR) analyses demonstrated that the polysaccharide chain structure of SGFs was not affected by H_2_O_2_ degradation, but alterations were detected at the peak positions of some functional groups. In vitro study showed that SGFs significantly exerted a stronger antibacterial activity against *V. parahaemolyticus* and *V. harveyi* than NSG, which might be due to the low molecular weight and higher sulfation properties of SGF. SGF disrupted the bacterial cell membrane, resulting in leakage of intracellular biological components, and subsequently, cell death. Taken together, this study provides a basis for the exploitation and utilization of low-molecular-weight sulfated galactans from *G. fisheri* to prevent and control the shrimp pathogens.

## 1. Introduction

Many shrimp-producing countries have reported disease outbreaks that greatly affect shrimp productivity, leading to widespread socioeconomic losses [1]. *Vibrio parahaemolyticus* is an emerging pathogen that causes unusual acute mortality within 35 days after stocking and causes early mortality syndrome (EMS) or acute hepatopancreatic necrosis syndrome (AHPNS) in Pacific white shrimp and black tiger shrimp [2]. The wide distribution of *V. harveyi* in the shrimp farming environment causes early larval mortality, resulting in enormous losses in production and marketing [3,4]. Consequently, the control and prevention of *V. parahaemolyticus* and *V. harveyi* is an essential component of the shrimp culture industry.

Antibiotics are vital for the prevention and treatment of bacterial infections in shrimp. The use of antibiotics over time has led to an increase in the number of resistant strains, causing an imbalance in ecosystems, compromised immune systems, and even resulting in the occurrence of antibiotic residues in aquatic animals [5]. To ensure that food-producing shrimp are sustainable and safe for human consumption, alternative antibiotic treatment methods are required. Studies have shown that natural products such as chitosan and essential oils from microbes, red seaweed, plants, and animals exhibit capability to control bacterial infections in white shrimp and tilapia [6,7,8,9].

The Food and Agriculture Organization (FAO), in 2021 [10], reported that about 96 percent of the total seaweed production was concentrated in East Asia and Southeast Asia. In Thailand, seaweed productions are mainly from *Gracilaria*, *Hypnea*, *Porphyra*, *Acanthopora*, and *Caulerpa* [11]. Among these, the red seaweed *Gracilaria* is considered the most important species, which is intensively cultured and mainly used for human consumption and agar production [12,13]. The discovery of other uses of seaweed and its by-products besides food applications, including nutraceuticals and pharmaceuticals [14,15], have contributed to the exponential demand for seaweed and their derivatives, and the commercial expansion of seaweed farms.

Sulfated polysaccharides (SPs) extracted from *Gracilaria fisheri* have proven antiviral properties [14] and possess a broad range of bioactivities [16]. Several studies have shown that the bioactivities of polysaccharides are dependent on the molecular weight and the composition of structural units such as monosaccharides, linkage patterns, branching features, degree of polymerization, and sequence of sugar units [17,18]. Hence, the functionality and bioactivity of polysaccharides can be potentially enhanced through degradation of their molecular integrity [19]. H_2_O_2_ is a chemical degradation method proven to be an effective alternative for polysaccharide degradation [20] because it is easily accessible and simple to use. It produces highly reactive -OH radicals that can enhance the degradation process and is eco-friendly, subsequently decomposing into water and oxygen [21,22]. Furthermore, polysaccharide degradation by H_2_O_2_ has been demonstrated to have potent antibacterial activity against various gram-positive and gram-negative bacteria [23,24].

In this study, we aimed to modify native sulfated galactans from *G. fisheri* (NSG) using the H_2_O_2_ method of oxidative degradation to obtain small molecular weight (MW) fragments of NSG, followed by structural characterization and evaluation of antibacterial activity. The results of this study provide a possible utilization of sulfated galactans from red seaweed as an alternative treatment for the prevention of *V. parahaemolyticus* and *V. harveyi* infections.

## 2. Results

### 2.1. Physicochemical Properties of SGF

The physicochemical parameters of SGF and NSG including pH, MW, polydispersity (PD), carbohydrate content, sulfate content, and degree of sulfation (DS) are presented in Table 1.

NSG treated with different concentrations of H_2_O_2_ (0.4%, 2%, and 10%) produced degraded MW products of SGF0.4, SGF2, SGF10 with yields of 7.0 ± 0.3%, 2.12 ± 0.1%, and 1.28 ± 0.1%, by NSG weight, respectively. The pH values of SGF0.4, SGF2, and SGF10 were 5, 4, and 4, respectively, which were lower than NSG (pH 6). Analysis of the carbohydrate content revealed there was no significant difference (*p* > 0.05) for NSG (66.95 ± 0.72%) and SGF (62.88 ± 1.17%, 58.97 ± 0.61%, and 58.40 ± 3.38%, respectively, *w*/*w*). The sulfated content of SGF (12.02 ± 0.12%, 12.28 ± 0.92%, and 12.33 ± 0.51%) was significantly higher than that of NSG (9.95 ± 0.19%). The GPC profile of each polysaccharide fraction is shown in Figure S1 (Supplementary Material). GPC analysis indicated an average MW for SGF0.4, SGF2, and SGF10 of 115.76, 3.79, and 3.19 kDa, respectively, which was significantly lower than that of NSG (228.33 kDa). Polydispersity values < 1.2 are regarded as narrow disperse of the compounds, indicating that the polysaccharides SGFs are nearly homogeneous to the monomer unit.

### 2.2. Structural Characterization and Morphology of SGFs

**Figure 1:**
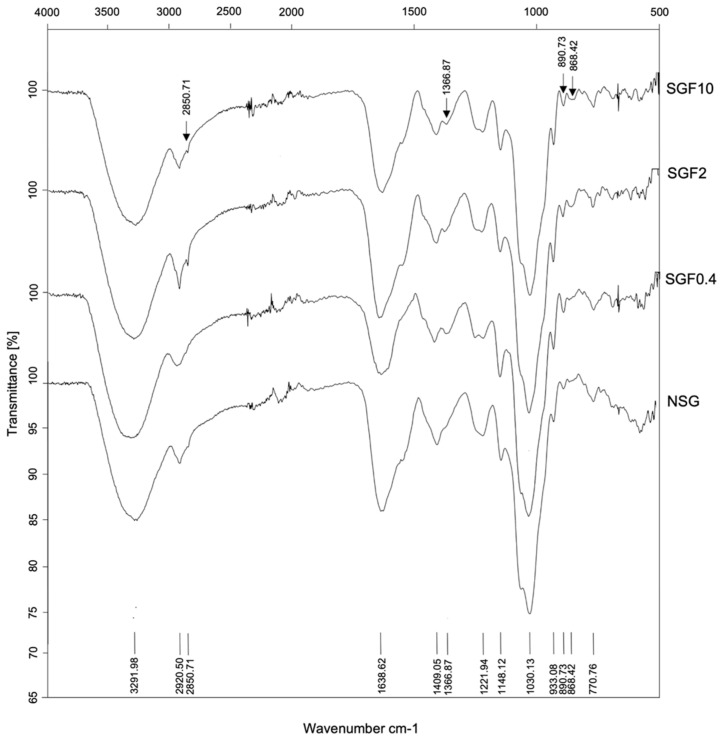
FTIR–ATR spectra of NSG and SGFs.

The FTIR–ATR spectra of NSG and SGFs are shown in Figure 1, and the assignments of the characteristic bands from the FTIR–ATR are given in Table 2. Sulfated polysaccharides typically have broad and strong absorption peaks in the range of 400–4000 cm^−1^. The peak at 3292 cm^−1^ indicates the presence of -OH stretching vibrations in NSG and SGFs. The peaks between 2840–2950 cm^−1^ indicate both symmetric and asymmetric CH stretching vibrations in the polysaccharides of NSG and SGFs [25]. Absorbance at 2850 cm^−1^ detected in SGF2 and SGF10 suggests that H_2_O_2_ degradation has produced opened symmetric CH groups in both SGFs.

Interestingly, the 1638 cm^−1^ peak, which corresponds to COO- antisymmetric stretching vibration, and the 1409 cm^−1^ peak, which corresponds to C=O symmetric stretching vibration [26], were decreased in both SGFs. As expected, carbohydrate structure vibrations were detected in both the NSG and SGFs. Absorbance peaks at 1148, 1030, and 933 cm^−1^ correspond to C-OH, C-C, and C-O-C of the galactan’s structure [27,28].

However, 1148 cm^−1^ was slightly weaker and 933 cm^−1^ was slightly stronger in SGFs compared with NSG. The peaks at 1366, 1221, 890, 857, and 770 cm^−1^ indicate the presence of sulfate ester groups [14]. The peak at 1366 cm^−1^, attributed to vibration of the sulfate ester, is seen in SGFs, which negatively correlates to the absorbance peak at 1221 cm^−1^, attributed to vibration of S=O (sulfate group). Peaks at 890 and 857 cm^−1^ are attributed to vibrations of galactose-6 sulfate and galactose-4 sulfate and are seen to be higher in SGF2 and SGF10, indicating the enhancement of sulfate ester by H_2_O_2_ degradation. In addition, the peak at 770 cm^−1^, attributed to vibration of C-O-S (sulfate group), was detected in NSG and SGFs. The results suggest that H_2_O_2_ degradation modification affects the sulfate ester of SGFs.

**Figure 2:**
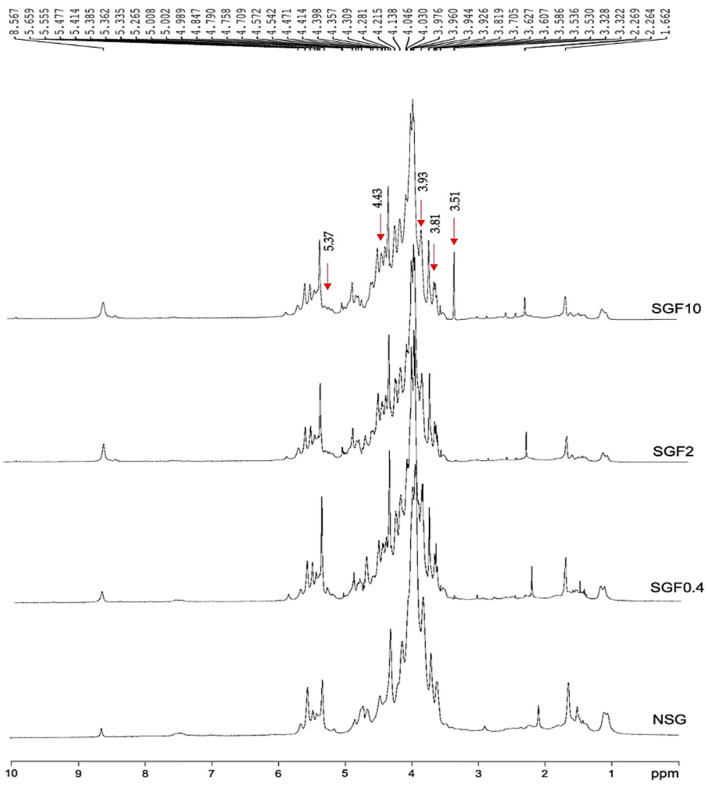
^1^H-NMR spectra of NSG and SGFs.

The ^1^H–NMR spectra for NSG and SGF are shown in Figure 2. Resonance of α- and β-anomers can be deduced from the chemical shift signals at 4.9–5.6 ppm and 4.41–4.81 ppm [29]. The chemical shift signals at 4.55, 3.63, 3.81, 4.14, 3.71, 3.83 ppm correspond to β-D-galactose unit, whereas signals at 5.16, 4.14, 4.54, 4.67, 4.57, 4.22 correspond to 3,6-α-L-anhydrogalactose unit, which were observed in NSG and SGFs. The chemical shift signal at 5.34 ppm indicates the presence of (1→4) α-L-galactose-6-sulfate in NSG and all the SGFs. The chemical shift signal at 5.34 ppm indicates the presence of (1→4) α-L-galactose-6-sulfate (L6S) in NSG and all the SGFs. The signals at 5.37, 3.81, and 3.93 ppm of all the SGFs are attributed to L6S-1, L6S-2, and L6S-3, respectively. In addition, the chemical shift signal at 4.43 ppm in all the SGFs corresponds to β-D-galactose-linked to α-L-galactose-6-sulfate. Interestingly, the chemical shift signal at 3.51 ppm, which was observed in SGF10, corresponds to H-6 of β-D-galactose-linked to α-L-galactose-6-sulfate residue [30].

**Figure 3:**
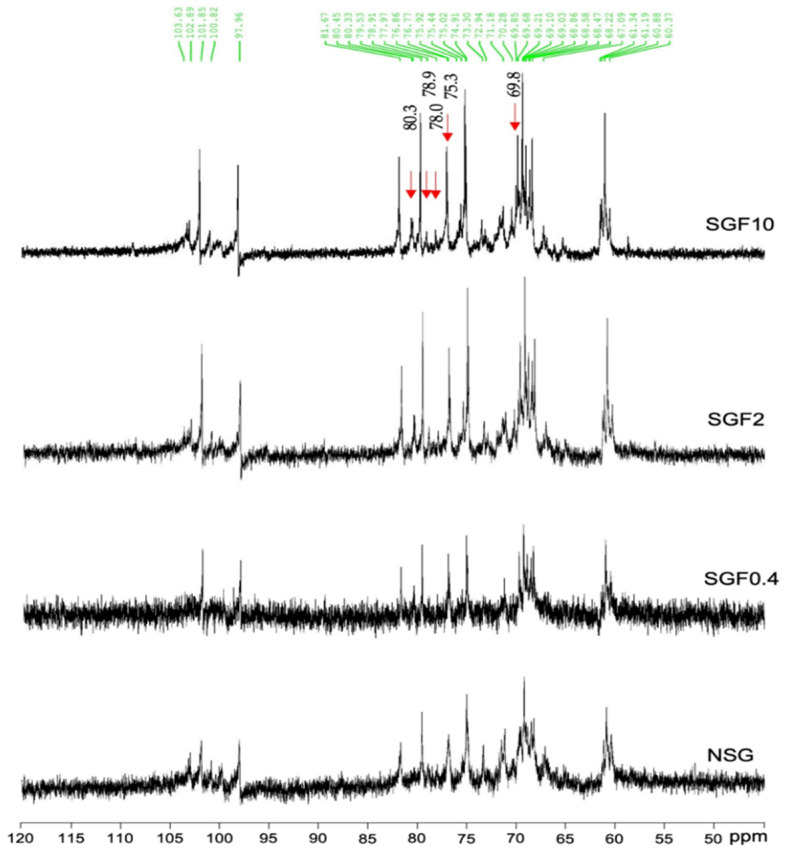
^13^C-NMR spectra of NSG and SGFs.

The ^13^C–NMR spectra of NSG and SGF are shown in the Figure 3. According to the literature, the 90–110 ppm chemical shift signals correspond to the anomeric carbon region [29,31]. The presence of β-D-galactose and 3,6-α-L-galactose in NSG and all the SGFs are inferred from the occurrence of chemical shift signals at 102.9 ppm and 98.0 ppm. The chemical shift signal at 103.7 ppm indicated the presence of β-D-galactose linked to α-L-galactose 6-sulfate [30] and the chemical shift signals at 101.9 ppm, 81.7 ppm, and 79.5 ppm indicated the presence of (1→3) β-D-galactose (1→4) α-L-galactose, 4-α-D-anhydrogalactose, and 2-O-Me-3,6-anhydrogalactose, which were observed in NSG and all the SGFs. The chemical shift signal at 78.9 ppm indicated the presence of (1→4) α-L-galactose-6-sulfate, which was observed only in SGF2 and SGF10. Moreover, the chemical shift signal at 76.9 ppm indicating the presence of 3-β-D-Gal-4-sulfate was also observed in NSG and all the SGFs. However, the chemical shift signals at 80.3, 78.0, and 75.3 ppm attributed to C3, C4, and C5 of D-galactose-4-sulfate residues and at 69.8 attributed to C2 of α-L-galactose-6-sulfate residue were observed in SGF2 and SGF10.

**Figure 4:**
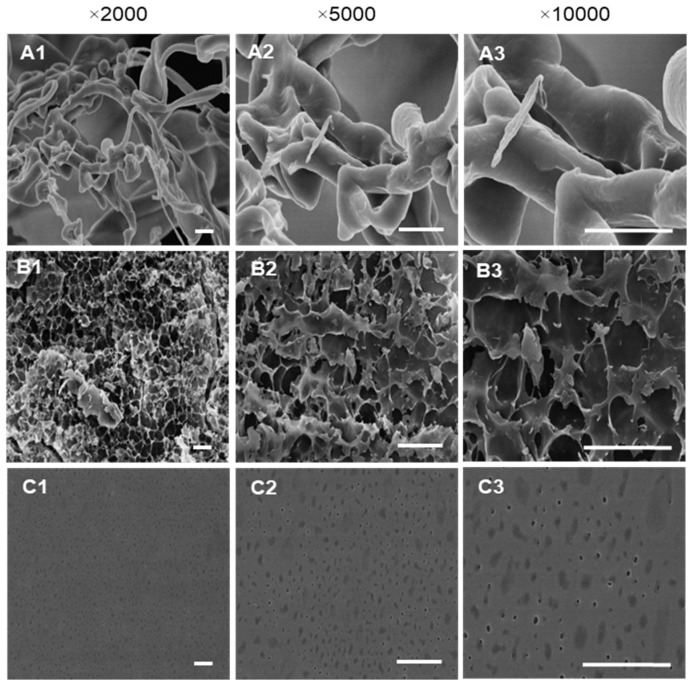
SEM micrographs of NSG (**A1**–**A3**), SGF0.4 (**B1**–**B3**), and SGF10 (**C1**–**C3**) at different magnifications (×2000; 5000; 10,000; 10 kV). Scale bar = 5 µm.

Scanning electron microscopy (SEM) was performed to compare the surface of NSG with SGFs. The results revealed that NSG was more compact with a smooth dense surface and distributed as individual filamentous particles (Figure 4(A1–A3)). SGF0.4 exhibited loose irregular fragmentary aggregates with a rough, porous surface (Figure 4(B1–B3)), while SGF10 had a sheetlike appearance with a smooth surface and clear pores (Figure 4(C1–C3)). SGF2 showed a similar morphology to those of SGF10 (data not shown). The SEM clearly showed that the compact structure of NSG was degraded to small molecule polysaccharides. 

### 2.3. Degraded Fractions of SG Exhibited Greater Antimicrobial Activity 

**Figure 5:**
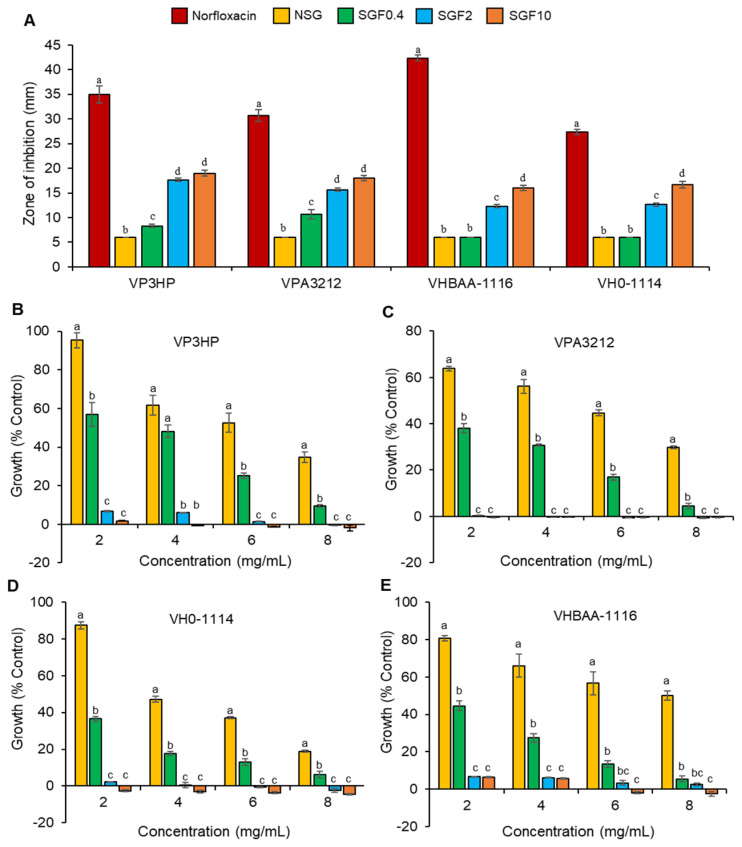
Antimicrobial activity of SGFs. Zone of inhibition results of VP3HP, VPA3212, VHBAA-1116, and VH0-1114 (**A**). Growth inhibition on the bacterial strains VP3HP (**B**), VPA3212 (**C**), VH0-1114 (**D**), and VHBAA-1116 (E), using a liquid turbidity assay with an incubation for 24 h. Results are expressed as a mean of three replicates ± SE. Mean values with different superscripts for each bacterium (**A**) and each concentration (**B**–**E**) indicate a significant difference (*p* < 0.01).

The antimicrobial activities of the SGFs were investigated using a disc diffusion assay against *V. parahaemolyticus* (VP3HP and VPA3212) and *V. harveyi* (VH0-1114 and VHBAA-1116). The results showed that SGF0.4, SGF2, and SGF10 significantly increased (*p* < 0.01) the diameter of the inhibition zone in VP3HP and VPA3212 compared with NSG. However, with VH0-1114 and VHBAA-1116, the SGF2 and SGF10 inhibition was greater than NSG and dose dependent (Figure 5A). The turbidity assay showed a dose-dependent decrease in bacterial proliferation of NSG and SGF0.4 for all bacterial strains (Figure 5B–E). At similar concentrations, SGF0.4 produced a stronger antibacterial activity against all tested strains than NSG. Increasing the concentration of SGF0.4 to 8 mg/mL suppressed the proliferation of all bacterial strains, while treatment with SGF2 and SGF10 suppressed all bacterial proliferation at a lower concentration of 2 mg/mL (Figure 5B–E).

The minimum inhibitory concentration (MIC) and minimum bactericidal concentration (MBC) values of NSG for all bacterial strains were 20 and 25 mg/mL, respectively. SGF0.4 had a lower MIC but similar MBC values to NSG. SGF2 and SGF10 had significantly lower MIC and MBC values compared with NSG and SGF0.4 (Table 3). The EC_50_ values of NSG and SGF0.4, SGF2, and SGF10 against the tested bacteria are shown in Table S1 (Supplementary Material).

### 2.4. Degraded Fractions of SG Inhibited the Growth of V. parahaemolyticus and V. harveyi

**Figure 6:**
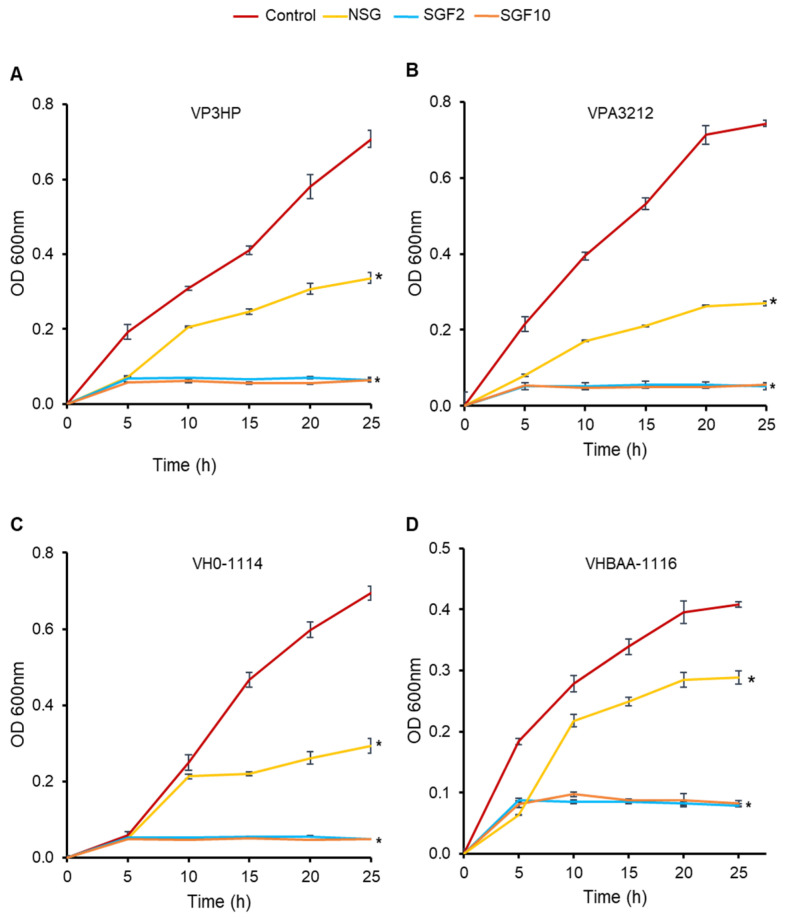
Effect of SGFs on the growth of bacterial strains VP3HP (**A**), VPA3212 (**B**), VH0-1114 (**C**), VHBAA-1116 (**D**). Control: Bacteria without treatment. Results are expressed as a mean of three replicates ± SE. * indicates a significant difference compared with the respective control (*p* < 0.01).

Since the MIC and MBC values of SGF0.4 were not much different from those of NSG, the subsequent experiments were then performed using SGF2 and SGF10 to compare with NSG. The time intervals for bacterial growth in the presence of NSG, SGF2, and SGF10 MICs were plotted to further validate their growth-inhibiting effect against VP3HP, VPA3212, VH0-1114, and VHBAA-1116 (Figure 6).

All bacterial strains grew normally in the control group, and the logarithmic growth stage was achieved within 10 h of incubation. When the bacteria were treated with the SG fractions (NSG, SGF2, and SGF10), they showed a substantially lower growth rate than that of controls. Moreover, after 5 h of incubation, no further growth of all the bacterial strains was observed in SGF2- and SGF10-treated groups while bacteria treated with NSG continued to grow, though at a lower rate than controls.

Pearson’s correlation coefficients analysis revealed that the antimicrobial activity of SGF was significantly (*p* < 0.01) positively correlated (R^2^ = 0.847) with H_2_O_2_ concentration and significantly (*p* < 0.01) negatively correlated (R^2^ = −0.902) with MW.

### 2.5. Degraded Fractions of SG Disrupted the Cell Membrane of V. parahaemolyticus and V. harveyi

**Figure 7:**
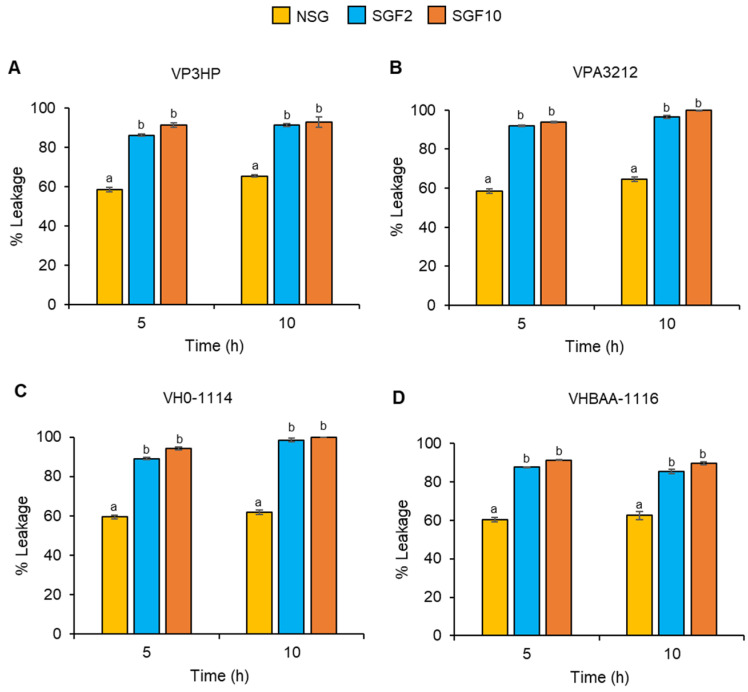
Leakage of cytoplasmic contents from *V. parahaemolyticus,* VP3HP (**A**) and VPA3212 (**B**), and *V. harveyi*, VH0-1114 (**C**) and VHBAA-1116 (**D**), after treating within MIC of NSG, SGF2, and SGF10. Results are expressed as a mean of three replicates ± SE. Different superscripts in each time frame indicate a significant difference (*p* < 0.01).

The membrane integrity of the bacteria was investigated to verify the ability of the degraded NSG fractions to damage the bacterial cell membrane. According to the growth-inhibiting results, treatment with SGF2 and SGF10 MICs for 5 h significantly decreased bacterial growth compared with NSG and SGF0.4. The membrane integrity was then determined at 5 h and 10 h of incubation. After 5 h exposure to the MIC of NSG, SGF2, and SGF10, the leakage of cytoplasmic content averaged 59.2%, 88.7%, and 92.6%, respectively. Further incubation of all the compounds for 10 h did not significantly induce more leakage (Figure 7).

**Figure 8:**
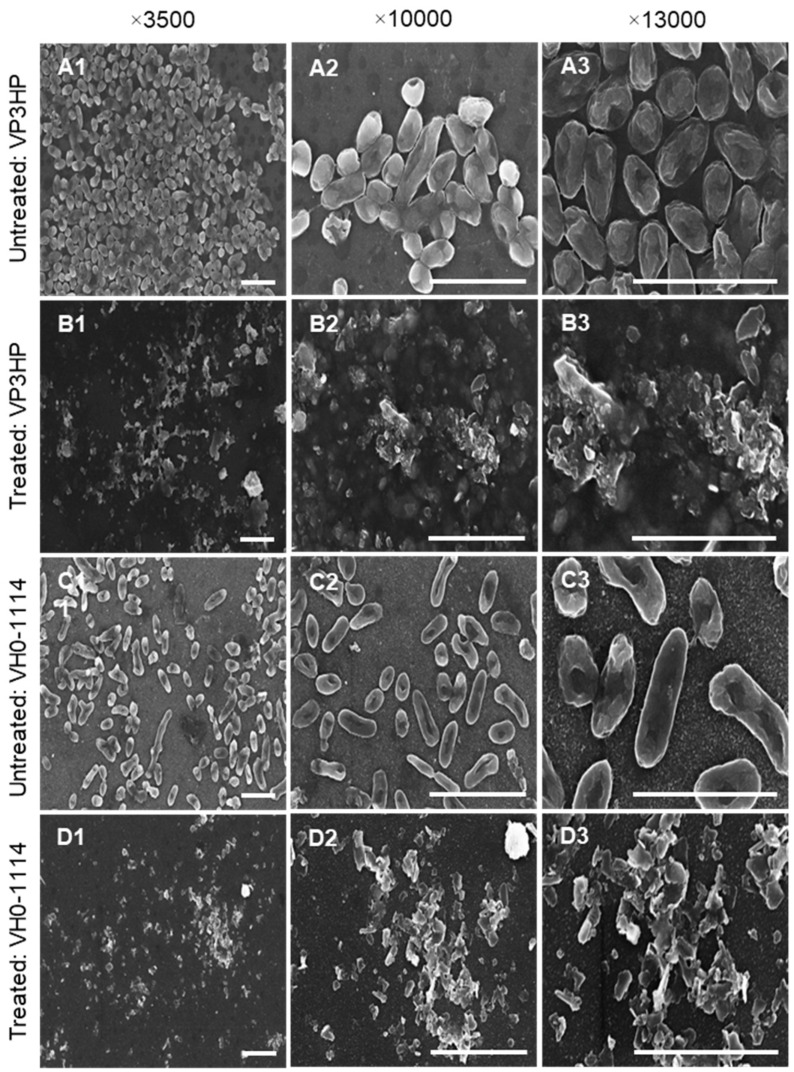
SEM micrographs of VP3HP-untreated (**A1**–**A3**), SGF10-treated (**B1**–**B3**), VH0-1114-untreated (**C1**–**C3**), and SGF10-treated (**D1**–**D3**) cells at different magnifications (×3500; 10,000; 13,000; 10 kV). Treatment was MIC concentrations for 5 h. Scale bar = 5 µm.

Scanning electron microscopy was used to investigate the potential damage to the cell membrane of *V. parahaemolyticus* (VP3HP) and *V. harveyi* (VH0-1114) by SGF10. SEM micrographs revealed that the normal morphology of VP3HP (Figure 8(A1–A3)) is a rod-shaped cell with a comparatively flat exterior surface and intact cell membrane. The normal VH0-1114 cell (Figure 8(C1–C3)) showed a regular, short rod-shaped morphology, with a smooth and intact cell surface. Following treatment of VP3HP and VH0-1114 cells with SGF10 MIC for 5 h, the cells became distorted, deformed, and shriveled shaped. Furthermore, many of the cells ruptured and the cytoplasm leaked out, resulting in blurring of cell edges Figure 8(B1–B3) and (D1–D3)).

## 3. Discussion

Sulfated polysaccharides (SP) possess a broad range of bioactivities [16]. However, the high molecular weight of many SPs limits some certain bioactivities and their pharmaceutical application. Numerous methods such as acid hydrolysis, oxidation, and enzymatic techniques have been used to degrade the polysaccharides, which have then been shown to enhance biological activity of the natural polysaccharides. Degradation by H_2_O_2_ oxidation is a commonly accepted method for polysaccharide modification and changing polysaccharide’s functional role [20]. Therefore, in the present study, the H_2_O_2_ oxidation method was used to hydrolyze the native sulfated galactans (NSG) extracted from red seaweed *G. fisheri* to obtain the degraded fractions (SGF) and evaluate their antibacterial activity against shrimp pathogenic bacteria.

Free radicals are primarily responsible for H_2_O_2_’s ability to reduce polysaccharide MW [32]. Hydroperoxide anion are easily broken down into highly reactive hydroxyl radicals. Generation of large quantities of the hydroxyl radicals allows them to easily access the glycosidic linkages and break the MW of polysaccharides, leading to their degradation [33]. Our study showed that the rate of NSG degradation into SGFs significantly depends on the concentration of H_2_O_2_. The high-molecular-weight NSG (228.33 kDa) hydrolyzed with 0.4%, 2%, and 10% H_2_O_2_ was degraded to notably lower molecular weight SGFs of 115.76, 3.79, and 3.19 kDa, respectively. In studies with high concentrations of H_2_O_2_, the resulting high acidity was found to enhance degradation efficiency, which might be due to a high quantity of hydroxyl radicals [34]. However, our study showed that increasing the concentration of H_2_O_2_ from 2% to 10% did not cause much further degradation. Previous research had reported that increasing the H_2_O_2_ concentration from 3% to 15% degraded the *G. lemaneiformis* SP (622 kDa) to 6.14 and 2.42 kDa, respectively [35]. This suggested that H_2_O_2_ at 2–3% may degrade high-MW sulfated polysaccharide to less than 10 kDa. H_2_O_2_ reduces the size of polysaccharides via its ability to produce potent oxidizing agents (hydroxyl radicals) that can break down the glycosidic linkages of polysaccharides [36], leaving the main chain structure of the polysaccharide unaltered [35]. This is consistent with our finding that H_2_O_2_ did not generate distinctive structural alterations of the main chain structure of SGFs but affected the side groups of the polysaccharides [37]. This is demonstrated by the similar profiles of FTIR–ATR spectra of NSG and SGFs, with differences only in the peak intensity. The NSG and SGFs revealed the−σ* and/or π−π* transitions in functional groups such as amine, carboxyl, carbonyl, and ester [38].

In addition, ^1^H- and ^13^C-NMR analyses were employed to investigate the SGFs’ structural configurations. The results also indicated that the β-D-galactose and 3,6-α-L-galactose units, the main chain structures of *G. fisheri* sulfated galactans [14], were observed and unaffected after H_2_O_2_ degradation. The appearance of increased sulfate ester chemical shift signals (at 5.37, 4.43, 3.93, 3.81, and 3.51 ppm by ^1^H- analysis and at 80.3, 78.9, 78.0, and 75.3 ppm by ^13^C-NMR analysis) in SGFs indicated the functional groups that were altered after H_2_O_2_ degradation [35,39]. It has been reported that the substitution of the sulfate groups on the polysaccharide backbone affects biological activity of the L configuration of 3,6 anhydro- derivatives of polysaccharides [40]. SGFs had a considerably (*p* < 0.01) higher degree of sulfation than NSG, which may be the result of free radical effects of H_2_O_2_ on the structure of polysaccharides [41]. SGF2 and SGF10 had an increase in sulfation at galactose-6-sulfate and galactose-4-sulfate, which may contribute to their superior antibacterial activity. This is consistent with a previous study, which showed that higher sulfate content in degraded SP from brown seaweed *Laminaria japonica* promotes antibacterial activity against *Escherichia coli* [42]. Moreover, SGF10 has a sheetlike structure with pores, which may allow for better interaction with the bacteria and subsequent increase in antibacterial activity. Our data agree with an earlier study, which showed that the sulfated derivatives have stronger antibacterial activity compared with the natural polysaccharides [43].

We tested the antibacterial activity of NSG and the degraded NSG against *V. parahaemolyticus* and *V. harveyi* and found that MIC of NSG (228.33 kDa) was as high as 20 mg/mL while MW SGF2 and those of SGF10 MIC’s were 4-16 times lower (1.25–5 mg/mL). In addition, bacterial growth in the presence of SGF2 and SGF10 MICs was completely suppressed at 5 h, while NSG-treated bacterial growth increased with time but at a lower rate than the control bacteria. We observed that SGF2 and SGF10 (3.19 and 3.79 kDa, respectively) at 2 mg/mL completely suppressed the proliferation of VP3HP, VPA3212, VH0-1114, and VHBAA-1116. However, SGF0.4 (115.76 kDa) required a higher concentration (8 mg/mL) for suppression. Statistically, the antimicrobial activity of different fractions of the sulfated galactans was significantly (*p* < 0.01) negatively correlated (R^2^ = −0.972) with MW. The overall data indicate that the smaller-sized SGF2 and SGF10 have a stronger antibacterial effect.

The previous studies have reported that the antimicrobial mechanism of action of sulfated polysaccharides is binding to the bacterial surface, which produces membrane breakdown, resulting in leakage of protein and vital nutrients and, ultimately, cell death [23,42]. In the present study, SGF10 (3.19 kDa), which have the least molecular weight and higher sulfation, exhibited a greater ability to disrupt the *V. parahaemolyticus* (VP3HP) and *V. harveyi* (VH0-1114) cell membrane, which has been witnessed. Our data agree with previous studies in which the lower molecular weight and higher sulfation of galactans from *Eucheuma serra* and *G. verrucosa*, and fucoidan from *Laminaria japonica* and *Sargassum polycystum*, produced a damaging effect on the cell membrane of *Escherichia coli, Staphylococcus aureus*, and *Pseudomonas aeruginosa* [42,44,45]. This suggests that the small-size SGF2 and SGF10 and the presence of negatively charged groups such as -COOH, -SO_4_ might be the important structural features to enhance bacterial membrane damage. However, it is still unclear through what mechanism negatively charged polysaccharides destroy the membrane structure of bacteria. We have hypothesized that SGF may interact with the positively charged molecules on bacterial cell surface such as some sugar moieties or cell membrane receptor, the interactions of which finally led to the loss of bacterial barrier function and cell lysis. Future studies should therefore aim to gain a comprehensive understanding of the mechanisms of interaction between SGF and bacterial cells, and, in particular, the mechanisms by which SGF disrupts the bacterial membrane.

## 4. Materials and Methods

### 4.1. Native Sulfated Galactans (NSG) and NSG Degradation 

Depigmented *G. fisheri* powder (10 g) was extracted in distilled water (40 °C) to obtain NSG as previously described [14]. Previously, we reported the structure of NSG (Figure S2, Supplementary Material) consisting of a linear chain of alternating units of 3-linked-β-D-galactopyranose (G) and 4-linked 3,6-anhydro-α-L-galactose (LA) or α-L-galactose-6-sulfate (L6S) with partial methylation (CH3) at C-2 of LA and C-6 of G, and sulfation of C-4 and C-6 of D-galactose units (G4S and G6S). HPLC analysis of NSG showed 90% purity [14]. NSG degradation was performed using H_2_O_2_ hydrolysis following the method described by Guo et al. [35]. Briefly, 10 mL of NSG (10 mg/mL) were heated to 40 °C for 15 min and H_2_O_2_ solution was quickly added to make various H_2_O_2_ concentrations at 0.4%, 2%, and 10%. The degradation reaction was allowed to proceed for 2 h at 90 °C. SG fractions with different molecular weights (SGF0.4, SGF2, and SGF10) were obtained. The NSG and SGF fractions were purified by anion exchange chromatography. Fractions were dissolved in distilled water, centrifuged at 10,000× *g* for 10 min, and the supernatant was collected for loading onto a DEAE-Sepharose fast flow column [46]. A DEAE-Sepharose fast flow bead solution was loaded into the column and then equilibrated with distilled water three times; then, the fraction solution was loaded onto the top of the column. The column containing the NSG/SGF solution was slowly washed with distilled water three times. The column was eluted with a stepwise ionic strength increment of NaCl (0.25–2.00 M) and a flow rate of approx. 0.5 mL/min. The eluted fractions containing NSG or SGFs were pooled, desalted using a dialysis membrane (NSG, 10,000 Da; SGF, 100–500 Da), freeze-dried, and kept at −20 °C until further use.

### 4.2. Sulfate and Carbohydrate Contents 

The sulfate content of the NSG/SGFs was determined using K_2_SO_4_ as a standard (0–0.2 mg/mL) [47]. Briefly, 1 mg of SGF was hydrolyzed in 250 µL of 0.5 mol/L HCl, vortexed, and the mixture was incubated for 3 h at 100 °C in a digital dry bath. The supernatant was transferred to a new microtube and centrifuged at 13,400× *g* for 15 min at room temperature (RT). Twenty microliters of the hydrolyzed sample were added to each well in a transparent polystyrene microplate that already contained 140 µL of HCl solution (0.5 mol/L HCl). Barium-chloride-gelatin reagent (40 µL) was added to the solution, mixed, and incubated at RT for 20 min. The absorbance was measured at 405 nm, and sulfate content in each sample was calculated using the regression equation (y = 0.2054x − 0.0019; R^2^ = 0.9849) from the standard K_2_SO_4_. The following equation was used to calculate degree of sulfation (DS):

DS = [(1.62 × S%)/(32 − 1.02 × S%)](1)

where DS is the average number of O-sulfate groups per sugar residue and S is the sulfate content.

The phenol sulfuric acid method was used to determine the carbohydrate content of NSG and SGFs, using D-galactose (0–1 mg/mL) as a standard [14]. Briefly, 100 µL SGF (1 mg/mL) was mixed with 100 µL 5% phenol, incubated at RT for 10 min, followed by an addition of 500 µL of concentrated sulfuric acid, vortexed, and left for 10 min at RT. The absorbance was measured at 490 nm, and the carbohydrate content was calculated using the regression equation (y = 4.2142x + 0.1279; R^2^ = 0.9841) from the standard D-galactose.

### 4.3. Molecular Weight Determination

The average molecular weight (MW) of SGF was determined by gel permeation chromatography (GPC, Agilent 1260 Infinity II, Santa Clara, CA, USA) [48]. SGF was dissolved (1 mg/mL) in deionized water and 20 µL of the sample solution was examined for each run. A Shimadzu LC-20AD with an LC-20A oven column and a RID-10A detector equipped with a TSKgel Guard PWH size exclusion column (mobile phase: deionized water) was used. The flow rate was 0.5 mL/min and the column temperature was 60.0 °C ± 0.1 °C. Dextran standards (DS-5000; DS-12,000; DS-50,000; DS-80,000; DS-150,000; and DS-270,000 (Sigma-Aldrich, St. Louis, MO, USA) were used to calibrate the column. The MW was determined using Shimadzu Class VP software to estimate the MW of the SGFs.

### 4.4. Fourier Transform Infrared (FTIR) Spectroscopic Analysis

Fourier transform infrared spectroscopy—attenuated total reflectance (ATR) spectra of the SGFs were acquired using an ALPHA FT-IR spectrometer with an ATR platinum diamond (Bruker, Hong Kong). Measurements from 500 to 4000 cm^−1^ (4 cm^−1^ resolution) were performed at room temperature (referenced against air) using 30 scans.

### 4.5. Nuclear Magnetic Resonance (NMR) Spectroscopy Analysis 

The deuterium oxide (D_2_O, 0.7 mL) was used to dissolve NSG and SGF (40 mg) in NMR tubes (5 mm diameter). ^1^H– and ^13^C–NMR spectra were recorded using a Bruker (AVANCE NEO, Billerica, MA, USA) 600 MHz NMR spectrometer at 80 °C. The chemical shifts of the ^1^H– and ^13^C–NMR were measured in parts per million (ppm) relative to the internal reference D_2_O at 4.7 ppm for ^1^H–NMR and at 0 ppm for ^13^C–NMR.

### 4.6. Bacterial Culture

*V. harveyi* (strains BAA-1116 and VH0-1114) and *V. parahaemolyticus* (strains 3HP and A3212) were kindly provided by the Center of Excellence for Shrimp Molecular Biology and Biotechnology (CENTEX Shrimp), Mahidol University, Thailand. The bacteria were cultured after inoculation of a single colony of bacteria in a 15-mL centrifuge tube containing 5 mL of Mueller Hinton broth (MHB, 3% NaCl). Every single isolate was incubated overnight in an incubator shaker at 30 °C and 200 rpm before being used in experiments.

### 4.7. Antibacterial Activity

Antibacterial activity was determined using the standard disc diffusion assay [49] and the turbidimetric measurement method [44]. For disc diffusion method, bacterial inoculums were spread on agar plates using a sterile glass spreader. Sterile Whatman No. 1 filter paper discs (6 mm diameter) were prepared and placed on the inoculated agar plate. The SGFs (100 µL, 5 mg/mL) were added to the discs. The cultures were then incubated upside down for 24 h at 30 °C. The same amount of distilled water was used as a negative control, and norfloxacin (50 µg/mL) was used as a positive control. The inhibition zone around the test paper disc indicated the absence of bacterial growth, which was reported to be positive. The absence of a zone was considered negative.

For the turbidimetric measurement, the final SGF concentrations of 2, 4, 6, and 8 mg/mL were prepared separately in a sterile microfuge tube containing 400 µL MHB, 500 µL SGFs, and 500 µL of previously prepared bacterial suspension (adjusted to McFarland standard of 0.5, which was equal to 1 × 10^6^ CFU/mL) and incubated for 24 h at 30 °C. The absorbance was measured at 600 nm using a microplate reader (VersaMax^TM^, Molecular Devices, San Jose, CA, USA).

### 4.8. MIC and MBC

The MIC and MBC of the SGFs were determined as described by the National Committee for Clinical Laboratory Standards [50], with slight modifications. The SGFs were dissolved in sterilized distilled water to a concentration of 25 mg/mL, and serial dilutions (15.0, 10.0, 5.0, 2.5, 1.25, 0.62, 0.31, and 0.15 mg/mL) were prepared for both MIC and MBC tests. A sterile microplate containing 100 µL of MHB was inoculated with 20 µL of a previously prepared bacterial suspension (1 × 10^6^ CFU/mL) and filled with 100 µL of SGF. Water and norfloxacin (50 µg/mL) were tested in a similar manner as the negative and positive controls, respectively. The plates were incubated at 30 °C for 24 h, and turbidity absorbance was measured at 600 nm. The MIC was determined as the lowest SGF concentration at which no bacterial growth was detected after 24 h incubation, whereas the MBC represented the lowest SGF concentration that showed no growth in the culture after incubation at 30 °C for 24 h. EC50 was calculated.

### 4.9. Bacterial Growth Curve

The effects of SGFs on the growth of bacterial pathogens were assessed using *V. parahaemolyticus* and *V. harveyi* [51]. The SG corresponding to MIC was dissolved in distilled water (40 µL) and added to 4 mL MHB medium. After mixing, 40 µL of the prepared bacterial culture (1 × 10^6^ CFU/mL) was added and incubated at 30 °C with shaking at 200 rpm. The absorbance of the bacterial sample (100 µL) was measured at 600 nm every 5 h for 25 h using a spectrophotometer (Eppendorf BioPhotometer, Hamburg, Germany). The mean and standard error of the mean (SE) for each sample were calculated for each time point of the growth curve.

### 4.10. Integrity of Cell Membrane 

When the cell membrane of bacteria is damaged, leakage of intracellular material occurs. Extruded bioendogenous UV-absorptive substances, which mainly include proteins, DNA, and RNA, can be measured to assess the integrity of cell membranes by detecting the UV absorbance at 260 nm [51]. The NSG/MIC and SGF/MIC (200 µL) were added to a 50-mL conical tube containing 10 mL of MHB medium. After mixing, 200 µL of the prepared bacterial culture (1 × 10^6^ CFU/mL) was added and incubated at 30 °C with shaking at 200 rpm. Control was the untreated bacteria. The bacterial sample (1 mL) was collected at 5 and 10 h of incubation, transferred into a tube containing 4 mL of distilled water, and centrifuged at 5000 rpm for 10 min. The absorbance of the supernatant containing the intracellular leakage was measured at 260 nm (Eppendorf BioPhotometer, Hamburg, Germany). The percent of intracellular material leakage was calculated using the following equation:

% Leakage = [(Control Absorbance/Treatment Absorbance) × 100](2)

### 4.11. Scanning Electron Microscopy 

Surface morphologies of the NSG and SGFs were determined using scanning electron microscopy (SEM, Hitachi SU-8010, Tokyo, Japan). An equal quantity of NSG and SGFs were kept on the surface of the stubs. These samples were prepared by platinum sputtering using a sputter coater and examined by a scanning electron microscope (Hitachi, Tokyo, Japan) with a 10 kV acceleration voltage. In addition, the effect of SGF10 on the cell membranes of *V. parahaemolyticus* (3HP) and *V. harveyi* (VH0-1114) was investigated following the previously described method [8]. Briefly, 4 mL of bacterial suspension (1 × 10^6^ CFU/mL) was mixed with SGF10 at MIC (3HP, 2.5 mg/mL and VH0-1114, 1.25 mg/mL) for 5 h at 30 °C and centrifuged at 5000 rpm for 10 min. Subsequently, cells were washed three times with 0.1 M sodium cacodylate buffer solution (pH 7.4) and centrifuged. The bacterial pellets (100 µL) were mounted over a poly L-lysine (0.01%) coated cover slip and dried for 24 h at RT. Dried bacterial cells were prefixed with 2.5% glutaraldehyde for 4 h and postfixed with 1% osmium tetra oxide for 2 h in 0.1 M sodium cacodylate buffer at 4 °C. The cells were serially dehydrated in ethanol (30, 50, 70, 80, 90, 95, and 100%), dried by critical point drying (CPD), and sputter-coated with a thin layer of platinum palladium. Finally, the cells were observed under a scanning electron microscope (Hitachi, Tokyo, Japan).

### 4.12. Statistical Analysis 

Data were expressed as the mean ± SE of at least three individual experiments. The statistical significance of the differences between the control and treatment groups was determined by one-way ANOVA using SPSS (version 26) followed by Tukey’s HSD test. Pearson’s correlation coefficients (r) were obtained for different properties of the SGFs. *p* < 0.05 was considered statistically significant. 

## 5. Conclusions

NSG degradation with H_2_O_2_ generated low-MW SGFs while maintaining the discrete structure of the polysaccharide chain. The low MW and high negative charge of SGFs produces superior antibacterial and bactericidal activities against *V. parahaemolyticus* (VP3HP and VPA3212) and *V. harveyi* (VH0-1114 and VHBAA-1116), unlike NSG, by interacting with the cell wall and membrane, which resulted in leakage of intracellular biological components. The EC50 value against the tested bacteria of SGF10 (about 1 mg/mL) was far less than that of NSG (about 5–7 mg/mL) (Table S1) suggesting the greater efficacy of the small MW SGF10 over NSG. Our findings highlight a potential opportunity for SGF10 to be used as an antimicrobial drug and provides a basis for their utilization in the prevention and control of shrimp pathogens. Furthermore, this study suggests that large-scale aquaculture of *G. fisheri* may be of use in the exploitation of a variety of food and nonfood enterprises.

## Figures and Tables

**Table 1:** Properties of NSG and SGFs.

Treatment	H_2_O_2_ Concentration (%)	MW (kDa)	PD	pH	Carbohydrate Content (%)	Sulfate Content (%)	DS
NSG	0	228.33 ^a^	1.29	6	66.95 ± 0.72 ^a^	9.95 ± 0.19 ^a^	0.74 ± 0.02 ^a^
SGF0.4	0.4	115.76 ^b^	1.08	5	62.88 ± 1.17 ^a^	12.02 ± 0.12 ^b^	0.99 ± 0.02 ^b^
SGF2	2	3.79 ^b^	1.04	4	58.97 ± 0.61 ^a^	12.28 ± 0.92 ^b^	1.03 ± 0.06 ^b^
SGF10	10	3.19 ^b^	1.19	4	58.40±3.38 ^a^	12.33 ± 0.51 ^b^	1.03 ± 0.06 ^b^

Results with the means of three replicates ± SE; mean values with the same small superscripts in the same column are not significantly different (*p* > 0.01). H_2_O_2_—hydrogen peroxide; MW—molecular weight; PD—polydispersity (Mw/Mn); DS—degree of sulfation (the average number of O-sulfate groups per sugar residue).

**Table 2:** FTIR bands of the functional groups presented in the NSG and SGFs.

Functional Group Characteristic Vibration (Wavenumber cm^−1^)	Fraction			
	NSG	SGF0.4	SGF2	SGF10
OH group (3291.98)	✓	✓	✓	✓
Asymmetric CH group (2920.05)	✓	✓	(+)	✓
Symmetric CH_2_ group (2850.71)	Non	Non	✓	✓
COOH of carboxylate group (1638.62)	✓	(-)	✓	(-)
C=O of carboxylate group (1409.05)	✓	(-)	(-)	(-)
Sulfate ester (1366.87)	Non	✓	✓	✓
S=O of sulfate group (1221.49)	✓	(-)	(-)	(-)
C-O of pyranose ring (1148.12)	✓	(-)	(-)	(-)
C-C of pyranose ring (1030.13)	✓	✓	✓	(-)
C-O-C of 3,6-anhydro-L-galactose (933.08)	✓	(+)	(+)	(+)
L-galactose-6 sulfate (890.73)	✓	(-)	(+)	(+)
D-galactose-4 sulfate (857.54)	✓	(-)	(+)	(+)
C-O-S of sulfate group (770.76)	✓	✓	✓	✓

Non—the vibration was not detected, (✓)—the vibration was detected, (+)—the vibration was increased compared with those of NSG, (-)—the vibration was decreased compared with those of NSG.

**Table 3:** MIC (mg/mL) and MBC (mg/mL) of SGFs against *V. parahaemolyticus* (VP3HP and VP3212) and *V. harveyi* (VH0-1114 and VHBAA-1116).

Bacteria	VP3HP		VPA3212		VH0-1114		VHBAA-1116	
Fraction	MIC	MBC	MIC	MBC	MIC	MBC	MIC	MIC
NSG	20	25	20	25	20	25	20	20
SGF0.4	15	25	15	25	15	25	15	15
SGF2	5	5	2.5	10	2.5	2.5	10	10
SGF10	2.5	2.5	1.25	1.25	1.25	1.25	5	5

## Data Availability

The data presented in this study are available on request from the corresponding author.

## Supplementary Materials

The following supporting information can be downloaded at: https://www.mdpi.com/article/10.3390/md20080469/s1, Figure S1: GPC chromatogram of NSG, SGF0.4, SGF2, and SGF10 showing the high MW of NSG was degraded to smaller MW SGFs by H_2_O_2._ oxidation (A). Average molecular weights of NSG and SGF obtained from H_2_O_2_ degradation (B), Figure S2: The structural feature of native sulfated galactans (NSG) from *G. fisheri* consists of 3-linked-β-d-galactopyranose (G) and 4-linked 3,6-anhydro-α-L-galactopyranose (LA) or α-l-galactose-6-sulfate (L6S) with partial methylaion (CH_3_) at C-2 of LA and C-6 of G, and presence of sulfation on C-4 and C-6 of d-galactopyranose units (G4S and G6S) [14]; Table S1: EC50 of NSG and SGF0.4, SGF2,SGF10 against VP3HP, VPA3212, VH0-1114, and VHBAA-1116.
