# Supplementary material for: Depolymerized Fractions of Sulfated Galactans Extracted from Gracilaria fisheri and Their Antibacterial Activity against Vibrio parahaemolyticus and Vibrio harveyi
Source: Mar Drugs. 2022 Jul 23;20(8):469. doi: 10.3390/md20080469 (PMC9394303; doi:10.3390/md20080469)
Supplement: Supplementary file 1 [file marinedrugs-20-00469-s001.zip › marinedrugs-1787845-supplementary.pdf]

Supplementary data

A

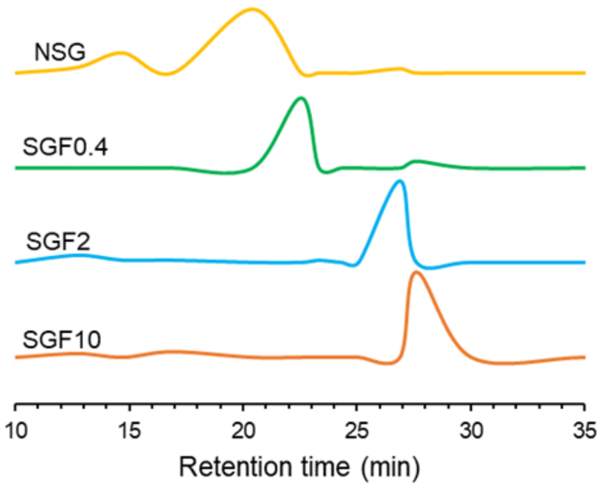

B

Fraction	Molecular mass (g/mol × 10^3, kDa)			Polydispersity	
	Mw	Mn	Mz	Mw/Mn	Mz/Mn
NSG	228.33	177.17	269.65	1.29	1.52
SGF0.4	115.76	107.58	123.06	1.08	1.14
SGF2	3.79	3.65	3.92	1.04	1.07
SGF10	3.19	2.67	3.51	1.19	1.32

Mw: weight-average molecular weight, Mn: number-average molecular weight, Mz: z-average molecular weight.

Figure S1. GPC chromatogram of NSG, SGF0.4, SGF2, and SGF10 showing the high MW of NSG was degraded to smaller MW SGFs by H_2O_2. oxidation (A). Average molecular weights of NSG and SGF obtained from H_2O_2 degradation (B)

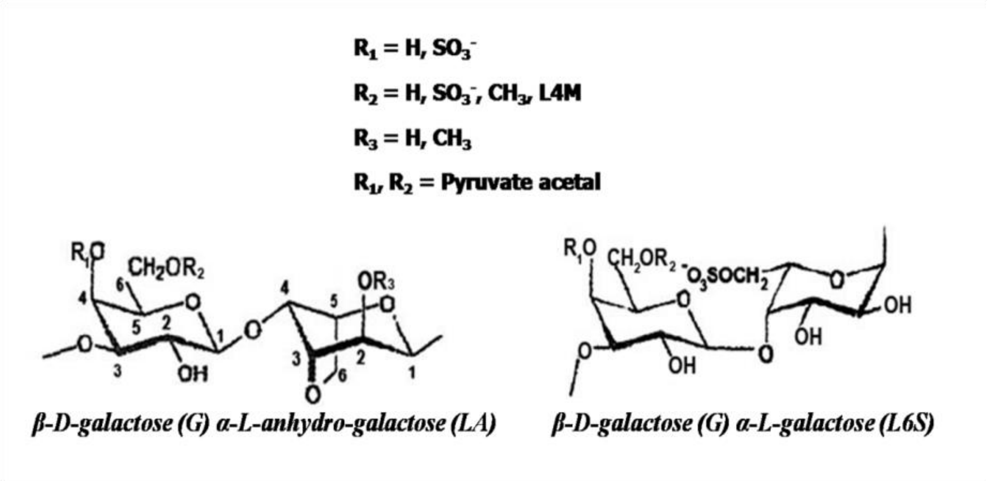

Figure S2. The structural feature of native sulfated galactans (NSG) from *G. fisheri* consists of 3-linked-β-D-galactopyranose (G) and 4-linked 3,6-anhydro-α-L-galactopyranose (LA) or α-L-galactose-6-sulfate (L6S) with partial methylaion (CH_3) at C-2 of LA and C-6 of G, and presence of sulfation on C-4 and C-6 of D-galactopyranose units (G4S and G6S) [14].

Table S1. EC50 of NSG and SGF0.4, SGF2,SGF10 against VP3HP, VPA3212, VH0-1114, and VHBAA-1116

Fraction	EC50 (mg/mL)			
	VP3HP	VPA3212	VH0-1114	VHBAA-1116
NSG	6.17	5.11	4.76	7.34
SGF0.4	3.80	2.87	2.55	2.92
SGF2	1.36	1.01	1.11	1.36
SGF10	1.06	0.98	0.88	1.33
